# Senescence Biomarkers CKAP4 and PTX3 Stratify Severe Kidney Disease Patients

**DOI:** 10.3390/cells13191613

**Published:** 2024-09-26

**Authors:** Sean McCallion, Thomas McLarnon, Eamonn Cooper, Andrew R. English, Steven Watterson, Melody El Chemaly, Cathy McGeough, Amanda Eakin, Tan Ahmed, Philip Gardiner, Adrian Pendleton, Gary Wright, Declan McGuigan, Maurice O’Kane, Aaron Peace, Ying Kuan, David S. Gibson, Paula L. McClean, Catriona Kelly, Victoria McGilligan, Elaine K. Murray, Frank McCarroll, Anthony J. Bjourson, Taranjit Singh Rai

**Affiliations:** 1 Personalised Medicine Centre, School of Medicine, Ulster University, Londonderry BT48 7JL, UK; 2 School of Health and Life Sciences, Teesside University, Campus Heart, Middlesbrough TS1 3BX, UK; 3 Western Health and Social Care Trust (WHSCT), Altnagelvin Area Hospital, Londonderry BT47 6SB, UK; 4 Belfast Health and Social Care Trust (BHSCT), Belfast City Hospital, Belfast BT9 7AB, UK

**Keywords:** senescence, chronic kidney disease, acute kidney injury, biomarker, machine learning

## Abstract

Introduction: Cellular senescence is the irreversible growth arrest subsequent to oncogenic mutations, DNA damage, or metabolic insult. Senescence is associated with ageing and chronic age associated diseases such as cardiovascular disease and diabetes. The involvement of cellular senescence in acute kidney injury (AKI) and chronic kidney disease (CKD) is not fully understood. However, recent studies suggest that such patients have a higher-than-normal level of cellular senescence and accelerated ageing. Methods: This study aimed to discover key biomarkers of senescence in AKI and CKD patients compared to other chronic ageing diseases in controls using OLINK proteomics. Results: We show that senescence proteins CKAP4 (*p*-value < 0.0001) and PTX3 (*p*-value < 0.0001) are upregulated in AKI and CKD patients compared with controls with chronic diseases, suggesting the proteins may play a role in overall kidney disease development. Conclusions: CKAP4 was found to be differentially expressed in both AKI and CKD when compared to UHCs; hence, this biomarker could be a prognostic senescence biomarker of both AKI and CKD.

## 1. Introduction

Acute kidney injury (AKI) is the abrupt cessation of kidney function due to heterogenous causes adding complication to critical illness [1]. Chronic kidney disease (CKD) is a global health burden causing a long-term reduction of kidney function resulting in CKD patients being at higher risk of morbidity, specifically from type 2 diabetes (T2DM) and cardiovascular disease (CVD) [2,3]. AKI and CKD are now believed to be intertwined conditions acting on similar pathways causing inflammation and fibrosis that lead to worse patient outcomes [4]. AKI-to-CKD progression can be characterised by a long-term reduction in kidney function measured using serum creatinine (sCr) and estimated glomerular filtration rate (eGFR) that does not return to baseline 3 months post-AKI [5]. Current estimates report that 25% of patients who experience AKI progress to CKD, with AKI increasing the likelihood of CKD diagnosis by a hazard ratio of 8.8 [6,7]. Furthermore, for those AKI patients who have CKD at the time of AKI (AKI-on-CKD), AKI leads to more rapid progression of CKD [8,9]. Progression of CKD is also common and can result in the need for renal replacement therapy (RRT) or transplant, yet progression rates differ for each patient, with some experiencing rapid progression of ≥5 mL/min/1.73 m^2^ per year [10,11,12].

Traditional clinical biomarkers such as sCr and urea lack the ability to highlight patients at increased risk of AKI progression or rapid CKD progression [13,14,15]. Previous research has aimed to identify proteins that stratify AKI and CKD patients’ progression risk; however, these studies often focused on specific types of CKD, used long follow-up periods, or they used proteins already associated with AKI or CKD diagnosis and involved in inflammation and fibrosis pathways [16,17,18,19]. Cellular senescence is the irreversible growth arrest that occurs in response to metabolic insults, oncogenic mutations, and DNA damage, whereby cells cease dividing and undergo distinctive phenotypic alterations [20]. Cellular senescence can also occur in terminally differentiated cells such as cardiomyocytes, neurons, and nephrons, characterised by the accumulation of senescent cells, which is hallmark of ageing and ageing pathologies [21,22]. Acute senescence is known to be beneficial, allowing for regeneration and repair in wound healing and immune clearance [23,24]. By contrast, chronic senescence, with the gradual accumulation of senescent cells over time, promotes deleterious effects accelerating deterioration and hyperplasia in ageing [25]. Furthermore, a wide array of intracellular and extracellular insults including pro-inflammatory mediators, proteotoxic stress, DNA damage, and mutations can accelerate senescence-related pathology [26].

In comparison to the general population, CKD patients experience accelerated ageing characterised by increased systemic inflammation, progressive vascular disease, muscle wasting, osteoporosis, and frailty, even before the onset of kidney failure [27]. Early vascular ageing (EVA) is characterised by vascular calcification, a cell-mediated process driven by alterations in vascular smooth muscle cells that has been shown to be a measure of biological age and a predictor of CKD and mortality [28]. This EVA causes the arterial walls of CKD patients to appear older than those of chronologically age-matched general-population individuals. This is due to the impact of chronic inflammation, reflecting premature adaptive changes because of repeated cellular insults, allostatic load, and an imbalance of anti-ageing and pro-ageing systems [29]. Although the mechanisms involved in EVA in CKD patients have not been fully elucidated, SASPs causing chronic inflammation appear to play a fundamental role in both initiation and progression [30,31]. In this study, we integrated proteomic targets associated with cardiovascular dysfunction, immune response, inflammation, and neurological impairment. The aim of this study was to quantify SASP protein expression [32] from patient plasma and investigate their effects in disease development and progression of AKI and CKD.

## 2. Materials and Methods

### 2.1. Participant Recruitment

Forty-three AKI patients (mean age = 61 years; 47% male) and 155 CKD patients (mean age = 59 years; 63% male) were recruited from Altnagelvin and Letterkenny University hospitals (Northwest of Ireland) as part of AKI outcome study (approved by Greater Manchester West Research Ethics Committee and NHS GRAMPIAN, North of Scotland Research Ethics Service; REC ref 18/NW/0348 and 18/NS/0067). AKI patient eligibility was determined by specific inclusion and exclusion criteria, where patients had to be ≥18 years of age at the time of recruitment and diagnosed with AKI stage 3 within the last 7 days in keeping with the AKI timeframe. Patients were excluded from the study if their AKI was less severe than stage 3, occurred more than 7 days prior to being able to be recruited, if they were receiving palliative care, or if they were unable to give informed written consent. AKI in this study was defined as an increase from baseline creatinine greater than three times. CKD patient eligibility was determined by specific inclusion and exclusion criteria where patient participants had to be ≥18 years of age at the time of recruitment and diagnosed with CKD of stage 2, 3a, 3b, or 4. Patients were excluded if diagnosed with stage 1 or stage 5 CKD, on dialysis, or had a previous kidney transplant. If informed consent could not be obtained, patients were excluded. CKD was defined in this study with a patient having an estimated GFR less than 60 mL/min. Due to lack of capacity, AKI patients were recruited within 7 days of AKI on ward, and CKD patients were recruited at outpatient clinics with disease stages ranging from 2–4 as per American Society of Nephrology CKD stages. AKI patients were followed up at 3 months post-recruitment using serum creatinine and eGFR data from the hospital electronic care record to establish progression status. One hundred multi-morbid patients who were null of kidney disease were used as controls (mean age 59.5 years; 47% male), selected to allow for age (*p* = 0.78), sex (*p* = 0.024), and BMI (*p* = 0.30) matching with AKI and CKD patients. The unhealthy controls (UHC) cohort included CVD patients (*n* = 49), diabetic patients (*n* = 43), and RA patients (*n* = 8). AKI progression was determined by AKI patients previously null of CKD remaining below the cut-off of 60 mL/min/1.73 m^2^ at the time of follow-up, or for AKI patients who had established CKD at the time of AKI, progression was determined by the CKD stage progressing to a more severe stage, e.g., from stage 2 CKD to stage 3a CKD.

### 2.2. Proteomic Analysis and Profiling Using Proximity Extension Assay

Levels of 535 unique proteins were measured using 5 µL of plasma per patient using the Proseek Multiplex proximity extension assay (PEA) (Olink Bioscience, Uppsala, Sweden) on the Olink Multiplex Plates: Cardiovascular panel II and Cardiovascular panel III, Immune response, Inflammation, Neuro exploratory, and Neurology. Following quality control (where over 80% of patients had a valid protein value), 476 unique proteins remained and were used for analysis. The use of Olink data collected for controls with chronic diseases allowed for 273 proteins to be compared across all three cohorts.

### 2.3. Statistical Analysis

Statistical analysis was conducted in R (v4.3.1) comparing base demographics of age, BMI, and gender across AKI, CKD, and UHC cohorts. AKI and CKD cohorts were tested independently with UHCs in a two-tail analysis. We expected biological differences between the cohorts analysed, and because of this, we employed Welch’s two-tailed *t*-test (*p*-value < 0.05) to account for unequal variances between AKI/CKD and UHCs. Code created in RStudio for statistical analysis within these cohorts will be publicly available on Github.

### 2.4. Machine Learning Predictions

Machine learning to determine protein predictive capabilities was carried out in R (v4.3.1), primarily using the caret package (v6.0-94) with additional supporting packages. Support vector machine (SVM) models were selected as the ideal model, as they possess the ability to select a linear kernel, allowing for accurate identification of single-protein biomarkers. Tenfold cross validation was used when building the model with hyperparameters such as cost being optimised through an iterative grid search function. Once models were generated, they were then fitted onto unseen data and set to predict, with the predictions being used to generate receiver operator characteristic (ROC) curves and area under curve (AUC) scores, which are used to determine predictive accuracy.

## 3. Results

### 3.1. Demographics for AKI Patients, CKD Patients, and Controls

Demographic and clinical data was measured for AKI, CKD, and controls with chronic disease (Table 1, Supplementary Table S1). All cohorts were age and BMI matched. Age and BMI were not found to be significant across AKI, CKD, and UHCs, confirming the cohort matching was a success and prevented confounding of the results. Gender was identified as significant within CKD, as 62.6% were male and 37.4% were female; however, this could be attributed towards the damaging effects testosterone plays versus the protective effects estrogen plays, which would agree with current literature, as males tend to progress to end-stage renal failure sooner than females.

### 3.2. MMP3, IL16, TNFRSF10C, CCL23, GDF15, TNFR1 and UPAR That Are Significantly Different between AKI Patients and Controls on Hierarchical Clustered Heatmaps

**Figure 1:**
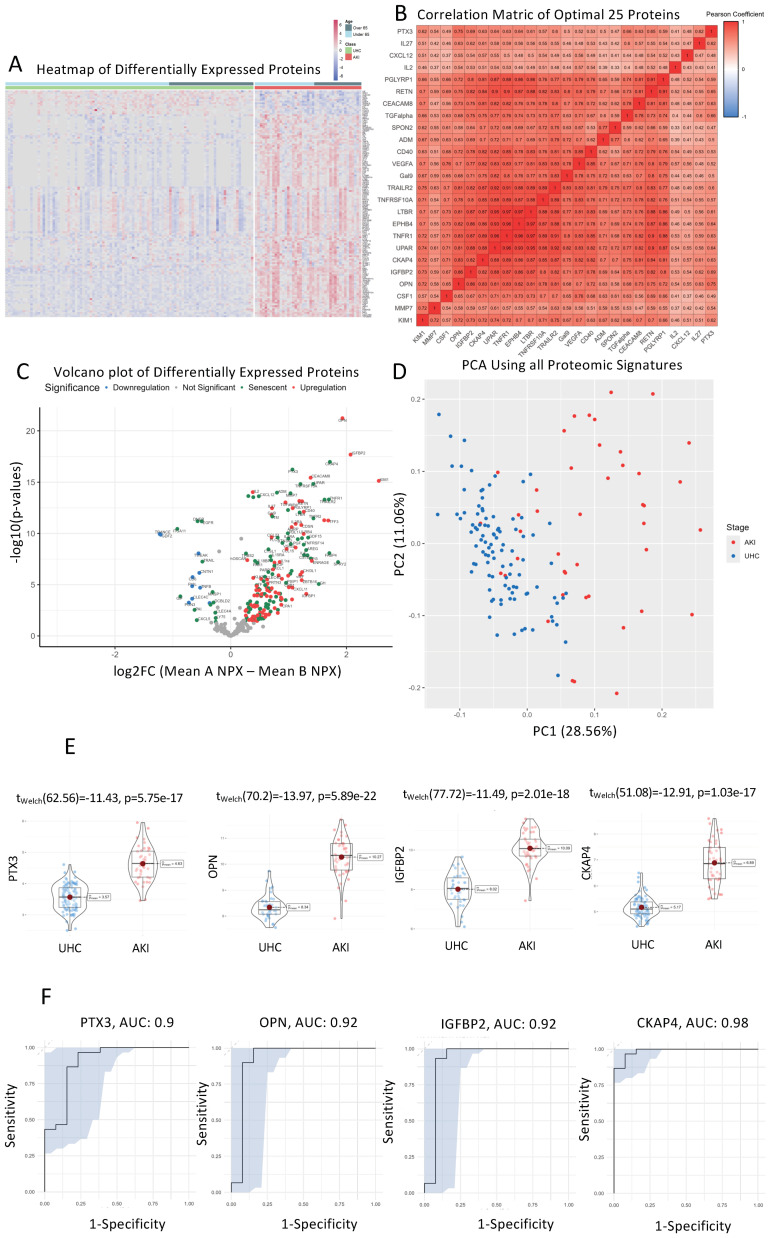
Senescence Proteins with Significant Differences in their Plasma Concentrations in AKI Patients vs. UHCs. (**A**). Heatmap of proteins that were significantly altered between AKI patients and UHCs using unsupervised clustering. Heatmap showing unsupervised clustering of the proteins that were significantly altered between AKI patients and UHCs. Clustering was conducted for gender, age, BMI, and cohort on the *x*-axis with protein clusters shown on the *y*-axis. Protein change is represented by coloured bars which use the UHC as the reference group. Red represents an upregulation in AKI patients and blue represents a downregulation in the AKI patients. Grey represents missing data. The darker the colour represents a greater change. Significance threshold was set at a log2FC of >0.25 & <−0.25, with a *p*-value < 0.01. (**B**). Correlation matrix of significantly altered protein between AKI patients and UHCs. A correlation matrix of the most significantly different proteins (*n* = 25) between AKI patients (*n* = 43) and UHCs (*n* = 100) was produced to highlight proteins that were strongly correlated. Colours on the graph represent the level of correlation, where red shows positive correlation and white shows no correlation. The darker the shade, the stronger the correlation, as can be seen in the colour shade bar below the correlation matrix. The protein quadrant reported in the analysis contains R^2^ values all greater than 0.8. (**C**). Volcano plot presenting differential expression analysis of altered proteins in AKI patients compared to UHCs. Volcano plot showing upregulated proteins are represented in red, and proteins that were downregulated are represented in blue, with green labelling indicating whether said protein is differentially expressed in senescence according to transcriptomic analysis of cell senescence. The *x*-axis on the volcano plot uses log2 (fold change) with the *y*-axis using −log10 (*p*-values), with a significance threshold being a log2FC of >0.5 & <−0.5, with a *p*-value < 0.05. (**D**). PCA analysis of AKI patients and UHCs. PCA was conducted on all proteomic signatures between AKI patients and UHCs to identify if there was clinical separation (Figure 2D). Each patient’s proteomic profile on the PCA is colour-coded to match their cohort criteria, red for AKI and blue for controls with chronic disease. Using PC1 and PC2 allowed for approximately 40% of variance to be explained and illustrates that there are two distinct groups formed between AKI patients and controls. (**E**). Violin plots of most significantly altered proteins between AKI patients and UHCs. Violin boxplots visualise individual proteomic differences between AKI patients and UHCs for the top four statistically significant proteins: CKAP4, PTX3, OPN, and IGFBP2 (*p* < 5.76 × 10^−17^). Protein expression was measured using NPX (Normalized Protein Expression) which is a log2 transformed metric quantified by OLINK proteomics. (**F**). Receiver operator curve analysis of the most significantly different proteins; TM, IL-2 and CKAP4, to identify AKI patients from UHC. ROC curves for each protein were generated from univariate SVM models to measure predictive capabilities of each protein. We used the AUC score as a metric to assess predictive accuracy: CKAP4 (AUC: 0.98), PTX3 (AUC: 0.90), IGFBP2 (AUC: 0.92), and OPN (AUC: 0.92).

A heatmap was created using unsupervised clustering of all proteins that were significantly different between AKI patients (*n* = 43) and controls (*n* = 100) (Figure 1A). Additionally, clinical classification and age were used to order patients and controls into over-65 years and under-65 years categories. The rationale behind accounting for age this way is to ensure that the differential senescence expression that we witness is not attributed to age specifically. For example, when we compare the cluster differences between AKI patients and UHC patients, we can clearly see that chronological age plays no part in the direction of expression of these proteins; rather, these proteins are specific signatures of AKI (Figure 1A). This suggests the SASP upregulation we see in AKI patients is not a consequence of ageing. Two major clusters of proteins were identified that showed upregulated and downregulated lists of proteins. Comparing the UHCs above the 65 years of age versus AKI patients above 65 years, there is a sub-group of upregulated SASP proteins in AKI such as MMP3, IL16, TNFRSF10C, CCL23, GDF15, TNFR1 and 2, and UPAR (*p* < 0.01). This suggests the age of patients is not confounding our senescence results.

### 3.3. TRAILR2, TNFRSF10A, LTBR, EPHB4, TNFR1, UPAR, CKAP4 and IGFBP2 Show Directly Proportional Correlation of Expression between AKI and UHC Cohorts

A correlation matrix was constructed using the most significant proteins that were different (*n* = 25) between AKI patients and controls (Figure 1B). Proteins that were most highly correlated are represented by a darker shade of colour, with red representing a directly proportional protein correlation and blue representing an inversely proportional protein correlation. An area of interest was decided upon by selecting the area which had a clustering of the most correlated proteins (Pearson’s correlation coefficient = 0.8). There were eight proteins in this area of interest, which included the following: TRAILR2, TNFRSF10A, LTBR, EPHB4, TNFR1, UPAR, CKAP4, and IGFBP2. This suggests these SASP proteins may share similar pathways specific to AKI when compared to UHCs.

### 3.4. Senescence Markers CKAP4, PTX3, UPAR, and TNFRSF10A Are Upregulated in AKI Patients Compared to Unhealthy Cohorts

All proteins were analysed using differential expression analysis and displayed in a volcano plot (Figure 1C). The volcano plot displays fold change (log2 fold change) versus significance (−log10 *p*-value) for AKI patients compared to UHCs. Senescence-specific proteins are coloured green. CKAP4, PTX3, UPAR, and TNFRSF10A were the most significantly upregulated proteins in AKI patients vs. UHCs (*p* < 1.50 × 10^−15^). EGFR, TRANCE, DNER, and ITGA11 were the most significantly downregulated proteins in AKI patients vs. UHCs (*p* < 1.16 × 10^−10^). SASP biomarkers comprise more than 50% (27/50) of all significantly different proteins.

### 3.5. Principal Component Analysis (PCA) of AKI Patients Compared to Controls

Principal component analysis (PCA) was conducted on all proteomic signatures between AKI patients and UHCs to identify if there was clinical separation (Figure 1D). Each patient’s proteomic profile on the PCA is colour coded to match their cohort criteria, red for AKI and blue for controls with chronic diseases. Using PC1 and PC2 allowed for approximately 40% of variance to be explained and illustrates that there are two distinct groups formed between AKI patients and controls. We can also see that the majority of class variance between AKI and chronic-diseased controls on the PCA is found on PC1, which suggests that the majority of variance that we see are based on proteomic differences between AKI and controls.

### 3.6. CKAP4, PTX3, OPN, and IGFBP2 Are the Most Differentially Expressed Senescent Proteins between AKI and UHC Cohorts

Violin boxplots were generated to visualise individual proteomic differences between AKI patients and UHCs for the top four statistically significant proteins: CKAP4, PTX3, OPN, and IGFBP2 (*p* < 5.76 × 10^−17^). Protein expression was measured using NPX (Normalized Protein Expression), which is a log2 transformed metric quantified by OLINK proteomics (Figure 1E). We can see from the proteomic violin boxplots that CKAP4, PTX3, OPN, and IGFBP2 are all upregulated in AKI patients compared to UHCs, suggesting their over-expression could be attributed to initial kidney injury in AKI patients.

### 3.7. Receiver Operator Characteristic Curves for Individual Proteins for AKI vs. Controls

ROC curves for each protein were generated from univariate SVM models to measure the predictive capabilities of each protein. We used the AUC score as a metric to assess predictive accuracy with each of the following: CKAP4 (AUC: 0.98), PTX3 (AUC: 0.90), IGFBP2 (AUC: 0.92), and OPN (AUC: 0.92). (Figure 1F). With the predictions from our SVM models on the test data, we generated ROC curves and calculated AUC scores by using the predicted probability values as decision thresholds then computed the corresponding sensitivities and specificities. With CKAP4, PTX3, IGFBP2, and OPN all being able to accurately differentiate between AKI and chronic disease controls, it suggests that there is distinct proteomic regulation within AKI patients, which these proteins are able to accurately capture, allowing for clear differentiation.

### 3.8. Senescent Proteins TM, CKAP4, and MMP7 Are Significantly Different between CKD Patients and Controls on Hierarchical Clustered Heatmaps

**Figure 2:**
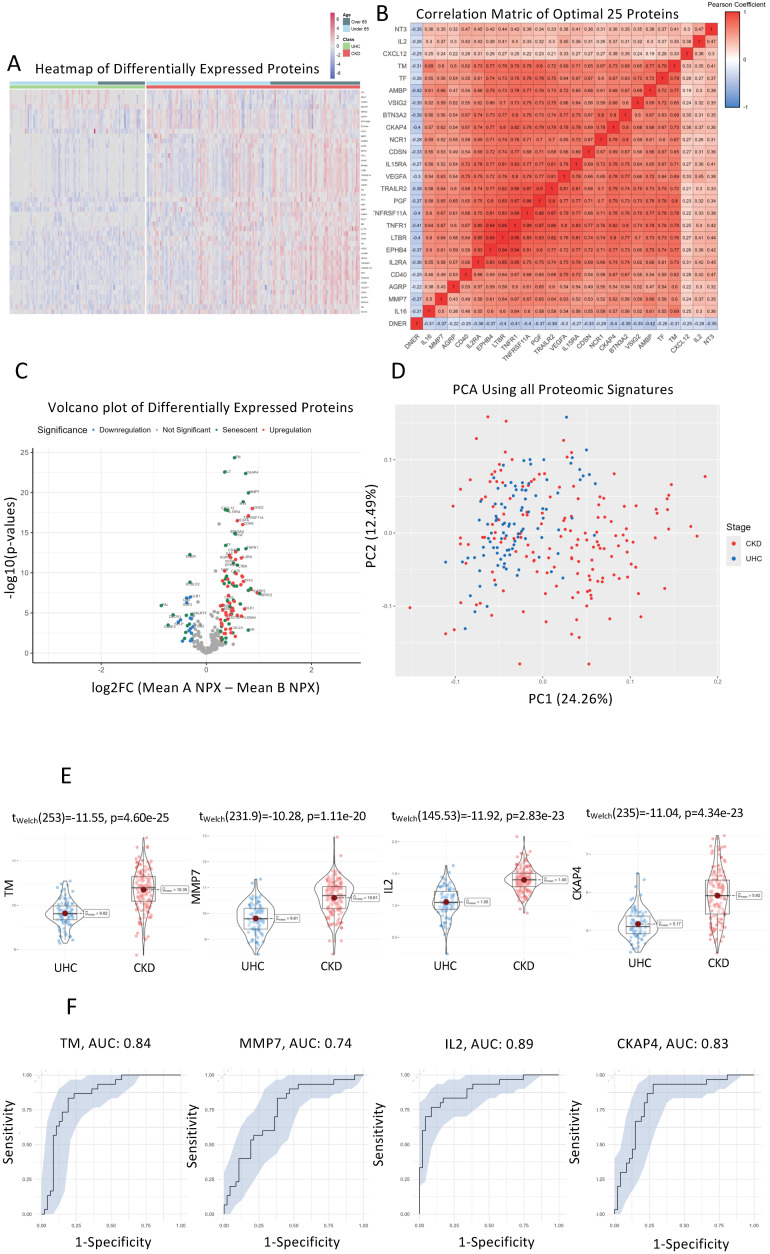
Senescence Proteins with Significant Differences in their Plasma Concentrations in CKD Patients vs. UHCs. (**A**). Heatmap of proteins that were significantly altered between CKD patients and UHCs using unsupervised clustering. Heatmap showing unsupervised clustering of the proteins that were significantly altered between CKD patients and UHCs. Clustering was conducted for gender, age, BMI, and cohort on the *x*-axis with protein clusters shown on the *y*-axis. Protein change is represented by coloured bars which use the UHC as the reference group. Red represents an upregulation in CKD patients and blue represents a downregulation in the CKD patients. Grey represents missing data. The darker the colour represents a greater change. Significance threshold was set at a log2FC of >0.25 & <−0.25, with a *p*-value < 0.01. (**B**). Correlation matrix of significantly altered protein between CKD patients and UHCs. A correlation matrix of significantly different proteins (*n* = 162) between CKD patients (*n* = 155) and UHCs (*n* = 100) was produced to highlight proteins that were strongly correlated. Colours on the graph represent the level of correlation where blue represents a positive correlation and red represents a negative correlation. The darker the shade, the stronger the correlation, as can be seen in the colour shade bar below the correlation matrix. The protein quadrant reported in the analysis contains R^2^ values all greater than 0.7. (**C**). Volcano plot presenting differential expression analysis of altered proteins in CKD patients compared to UHCs. Volcano plot showing upregulated proteins represented in red and proteins that were downregulated are represented in blue, with green labelling indicating whether said protein is differentially expressed in senescence according to transcriptomic analysis of cell senescence. The *x*-axis on the volcano plot uses log2 (fold change) with the *y*-axis using −log10 (*p*-values), with a significance threshold being a log2FC of >0.5 & <−0.5, with a *p*-value < 0.05. (**D**). PCA analysis of CKD patients and UHCs. Principal component analysis was conducted on all proteomic signatures between CKD patients and UHCs to identify if there was clinical separation. Using PC1 and PC2 allowed for approximately 37% of variance to be explained and illustrates that there are two distinct groups formed between CKD patients and controls. (**E**). Violin plots of most significantly altered proteins between CKD patients and UHCs. Violin boxplots visualise individual proteomic differences between CKD patients and UHCs for the top four statistically significant proteins: TM, CKAP4, NT3, and MMP7 (*p* < 1.12 × 10^−20^). Protein expression was measured using NPX (Normalized Protein Expression), which is a log2 transformed metric quantified by OLINK proteomics. (**F**). Receiver operator curve analysis of the most significantly different proteins; TM, IL-2, and CKAP4, to identify CKD patients from UHC. ROC curves for each protein were retrieved from univariate SVM models to measure predictive capabilities of each protein. We used the area under curve (AUC) as a metric to assess predictive accuracy with: TM (AUC: 0.84), MMP7 (AUC: 0.74), IL2 (AUC: 0.89), and CKAP4 (AUC: 0.83).

A heatmap was created using unsupervised clustering of all proteins that were significantly different between CKD patients and controls (*n* = 255) (Figure 2A). Additionally, clinical classification and age was used to order patients and controls into over-65 years and under-65 years categories. The rationale behind accounting for age this way is to ensure that the differential senescence expression that we witness is not attributed to age specifically. For example, when we compare the cluster differences between CKD patients over the age of 65 and UHC patients over the age of 65, we can clearly see that chronological age plays no part in the direction of expression of these proteins; rather, these proteins are specific signatures of CKD (Figure 2A). Two major clusters of proteins were identified that showed upregulated and downregulated lists of proteins. Comparing the UHCs above the 65 years of age versus CKD patients above 65 years, there is a sub-group of upregulated SASP proteins in CKD such as TM, CKAP4, and MMP7 (*p* < 0.01). This once again suggests that the age of patients is not confounding our senescence results.

### 3.9. TNFR1, LTB3, EPHB4, and IL2RA Show Directly Proportional Correlation of Expression between CKD and UHC Cohorts

A correlation matrix was constructed using the most significant proteins that were different (*n* = 25) between CKD patients and controls (Figure 2B). Proteins that were most highly correlated are represented by a darker shade of colour, with red representing a directly proportional protein correlation and blue representing an inversely proportional protein correlation. An area of interest was decided upon by selecting the area which had a clustering of the most correlated proteins (Pearson’s correlation coefficient = 0.8). There were four proteins in this area of interest, which included TNFR1, LTBR, EPHB4, and IL2RA. This suggests these SASP proteins may share similar pathways specific to CKD when compared to UHCs.

### 3.10. Senescent Proteins TM, IL2, CKAP4, and MMP7 Are Significantly Differentially Expressed Senescent Proteins between CKD and UHC Cohorts

All proteins were analysed using differential expression analysis and displayed in a volcano plot (Figure 2C). The volcano plot displays fold change (log2 fold change) versus significance (−log10 *p*-value) for CKD patients compared to UHCs. Senescence-specific proteins are coloured green. TM, IL2, CKAP4 and MMP7 were the most significantly upregulated proteins in CKD patients vs. UHCs (*p* < 1.12 × 10^−20^). DNER, DCBLD2, GLB1 and CD6 were the most significantly downregulated proteins in CKD patients vs. UHCs (*p* < 1.37 × 10^−7^). SASP biomarkers comprise 50% (25/50) of all significantly different proteins.

### 3.11. Principal Component Analysis (PCA) of CKD Patients Compared to Controls

Principal component analysis (PCA) was conducted on all proteomic signatures between CKD patients and UHCs to identify if there was clinical separation (Figure 2D). Using PC1 and PC2 allowed for approximately 37% of variance to be explained and illustrates that there are two distinct groups formed between CKD patients and controls. We can also see that the majority of class variance between AKI and chronic-diseased controls on the PCA is found on PC1, which again suggests that the majority of variance that we see is based on proteomic differences between CKD and controls and is being captured effectively on that principal component.

### 3.12. TM, CKAP4, IL2, and MMP7 Are the Most Differentially Expressed Senescent Proteins between CKD and UHC Cohorts

Violin boxplots were generated to visualise individual proteomic differences between CKD patients and UHCs for the top four statistically significant proteins: TM, CKAP4, NT3, and MMP7 (*p* < 1.12 × 10^−20^). Protein expression was measured using NPX (Normalized Protein Expression), which is a log2 transformed metric quantified by OLINK proteomics (Figure 2E). We can see from the proteomic violin boxplots that TM, CKAP4, IL2, and MMP7 are all upregulated in CKD patients compared to UHCs. The magnitude of expression is not as large as AKI compared to controls, which is what we expect, as we are comparing a chronic disease to a chronic disease, as compared to acute injury in AKI. This also suggests that persistent over-expression of senescence biomakers could be linked to the pathophysiology of CKD.

### 3.13. Receiver Operator Characteristic Curves for Individual Proteins for CKD vs. Controls

ROC curves for each protein were retrieved from univariate SVM models to measure predictive capabilities of each protein. We used the area under curve (AUC) as a metric to assess predictive accuracy with each of the following: TM (AUC: 0.84), MMP7 (AUC: 0.74), IL2 (AUC: 0.89), and CKAP4 (AUC: 0.83) (Figure 2F). With the predictions from our SVM models on the test data, we generated ROC curves and calculated AUC scores by using the predicted probability values as decision thresholds, then computed the corresponding sensitivities and specificities. With TM, CKAP4, IL2, and MMP7 all being able to accurately differentiate between CKD and chronic disease controls, it suggests that there is distinct senescence-specific proteomic regulation within CKD patients which accurately captures this relationship, allowing for clear differentiation.

### 3.14. Network Analysis of Differentially Expressed Proteins in AKI Compared to Controls

**Figure 3:**
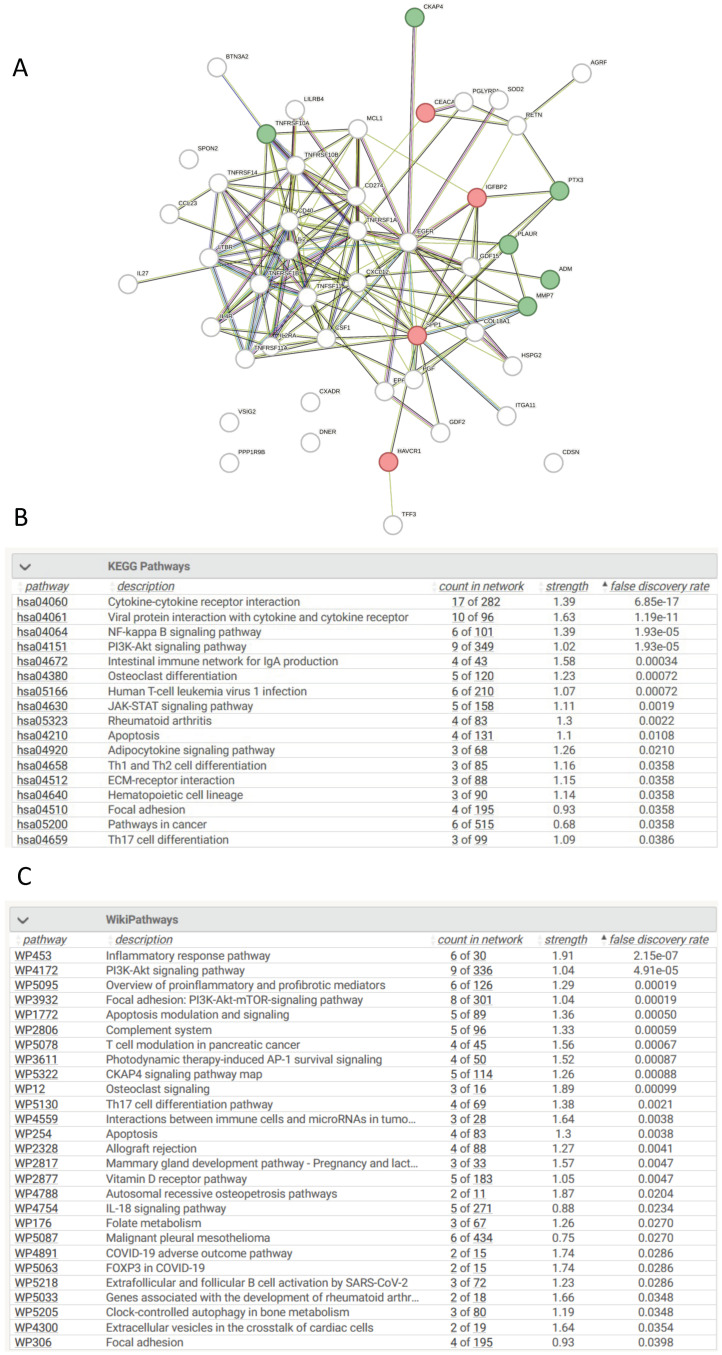
Pathway Analysis of Proteins with Significant Differences in their Plasma Concentrations in AKI Patients vs. UHCs. (**A**). Protein–Protein interaction network of significant proteins between AKI patients and UHCs. Protein–protein interaction networks generated using stringDB with the input being the corresponding gene symbol for each protein. The network comprises multiple nodes, each being a protein, which are interconnected to other proteins which visualise the interaction. Interactions have been found with literature research, scientific experiments, or data mining. Networks consisted of the top 50 proteins, with the top ten being labelled as green for senescence or red for non-senescence. (**B**). Enriched KEGG terms between AKI patients and UHCs. Biological enrichment statistics generated on stringDB, which provide the enriched KEGG terms based on specific proteins entered in the network. Count in network provides the number of genes present in the PPI map over the number of genes present in the signaling pathway. Strength provides a numerical value indicative of how strong the relationship between the proteins is, with the higher the number, the more impactful the relationship, and lastly, *p*-value of the network provides how statistically significant each KEGG pathway is enriched. (**C**). Enriched WikiPathway terms between AKI patients and UHCs. Biological enrichment statistics generated on stringDB, which provide the enriched WikiPathway terms based on specific proteins entered in the network. Count in network provides the number of genes present in the PPI map over the number of genes present in the signaling pathway. Strength provides a numerical value indicative of how strong the relationship between the proteins is, with the higher the number, the more impactful the relationship, and lastly, *p*-value of the network provides how statistically significant each WikiPathway pathway is enriched.

The top 50 statistically significant proteins between AKI and controls were queried in stringDB for network analysis with 48 of these proteins returning a hit search (Figure 3A). Various enriched signalling pathways were identified as statistically significant according to KEGG and Wiki pathways such as NF-kappa B, cytokine-cytokine receptor interactions, Inflammatory response pathways, and CKAP4 signalling pathways (*p* < 0.001) (Figure 3B,C). That these pathways identified as enriched between AKI patients and chronic disease controls is reassuring, as it suggests that the proteomic signatures that were queried are distinct regulators in AKI pathophysiology and could play an integral role during initial kidney injury.

### 3.15. Network Analysis of Differentially Expressed Proteins in CKD Compared to Controls

**Figure 4:**
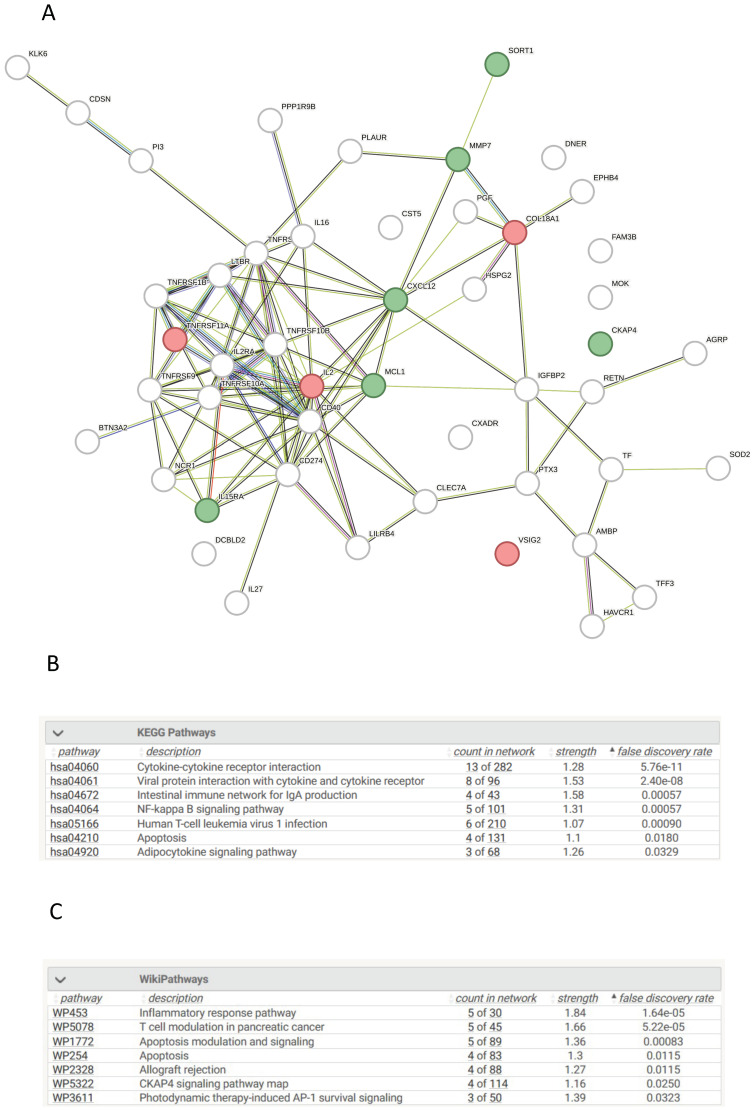
Pathway Analysis of Proteins with Significant Differences in their Plasma Concentrations in CKD Patients vs. UHCs. (**A**). Protein–Protein interaction network of significant proteins between CKD patients and UHCs. Protein–protein interaction networks generated using stringDB with the input being the corresponding gene symbol for each protein. The network comprises multiple nodes, each being a protein, which are interconnected to other proteins which visualise the interaction. Interactions have been found with literature research, scientific experiments, or data mining. Networks consisted of the top 50 proteins, with the top ten being labelled as green for senescence or red for non-senescence. (**B**). Enriched KEGG terms between CKD patients and UHCs. Biological enrichment statistics generated on stringDB, which provide the enriched KEGG terms based on specific proteins entered in the network. Count in network provides the number of genes present in the PPI map over the number of genes present in the signaling pathway. Strength provides a numerical value indicative of how strong the relationship between the proteins is, with the higher the number, the more impactful the relationship, and lastly, *p*-value of the network provides how statistically significant each KEGG pathway is enriched. (**C**). Enriched WikiPathway terms between CKD patients and UHCs. Biological enrichment statistics generated on stringDB, which provide the enriched WikiPathway terms based on specific proteins entered in the network. Count in network provides the number of genes present in the PPI map over the number of genes present in the signaling pathway. Strength provides a numerical value indicative of how strong the relationship between the proteins is, with the higher the number, the more impactful the relationship, and lastly, *p*-value of the network provides how statistically significant each WikiPathway pathway is enriched.

The top 50 statistically significant proteins between AKI and controls were queried in stringDB for network analysis with 48 of these proteins returning a hit search (Figure 4A). Various enriched signalling pathways were identified as statistically significant according to KEGG and Wiki pathways such as NF-kappa B, Cytokine-cytokine receptor interactions, Inflammatory response pathways, and CKAP4 signalling pathways (*p* < 0.05) (Figure 4B,C). These pathways, identified as enriched between CKD patients and chronic disease, although not as significant as within AKI, are still important. These pathways are enriched in CKD patients compared to patients with other chronic diseases, suggesting that these signatures are integral to kidney damage and disease. We can attribute the difference in significance to the immunological response differences between AKI and CKD, as we would expect AKI to have a more pronounced proteomic-inflammatory expression pattern due to initial kidney injury. Whereas CKD would have a lessened but persisting proteomic-inflammatory expression pattern.

## 4. Discussion

The aim of this study was to investigate the role of cellular senescence in AKI and CKD. To achieve this, we used OLINK plasma proteomics of kidney disease patients at various stages of renal dysfunction and compared it to SASP secreted by senescent cells. We found that levels of SASP cytoskeleton-associated protein 4 (CKAP4) and pentraxin-related protein (PTX3) were increased in AKI and CKD patients when compared to age-, sex-, and BMI-matched UHCs, suggesting that these senescence proteins play a role in AKI and CKD development. CKAP4 is involved in the maintenance of endoplasmic reticulum sheets and is already known to be associated with development of CKD and is a potential target for drug development for the treatment of kidney fibrosis [33]. Interestingly, network analysis identified the CKAP4 signalling pathway to be significantly enriched in both CKD and AKI cohorts (*p* < 0.05). Analysis in this study using principal component analysis (PCA) and heatmaps highlighted the marked difference between AKI patients and UHCs. These UHCs included age-matched CVD, diabetes, and RA patients without underlying kidney disease. This analysis included all measured biomarkers, with PCA showing that there was clear separation between AKI patients and UHCs yet lesser separation between CKD patients and UHCs. This shows that overall, when all biomarkers are included, CKD patients and UHCs may share similar dysregulated pathways. Similarly, heatmap analysis showed that AKI patients and controls were distinctly separated while CKD patients were less separated from controls, reinforcing the findings of PCA. This analysis is logical, as AKI patients are acutely seriously ill, often needing lifesaving clinical care as the body is responding to a severe attack. CKD patients who are chronically ill have a more similar biomarker response with unhealthy controls who have differing chronic conditions and inflammation, indicating that many chronic conditions react similarly in terms of the magnitude of biomarker upregulation/downregulation. However, two SASP proteins, CKAP4 and PTX3, were shown to be highly capable of highlighting both AKI and CKD patients for controls and to be of interest for further investigation.

CKAP4 is a type II transmembrane protein consisting of an N-terminal intracellular domain, a single transmembrane domain, and a C-terminal extracellular domain commonly situated in the endoplasmic reticulum and involved in many biological activities within the cell [34]. CKAP4 is well established as a biomarker of differing conditions, including playing a role in lung disease and in tumor formation and being upregulated in certain types of cancers [35]. Additionally, it is shown to be upregulated following ischemic injury, playing an important role in ventricular fibroblast activation [36], and in recent studies showing to be upregulated in both in vitro and in vivo mice CKD models in vascular smooth muscle cells, leading to vascular calcification by modulating YAP phosphorylation and MMP2 [33]. At present, however, no other clinical studies have identified an association between CKAP4 and AKI or CKD, making this study the first to do so. However, it is possible to hypothesise that with fibrosis and vascular calcification commonly present in both AKI and CKD, CKAP4 may play a role in driving these conditions leading to disease development. Furthermore, CKAP4 is shown to be involved in cell migration where cells with knockdown CKAP4 have a decreased level of cell migration [37]. As AKI is known to cause a reduction in cell migration, CKAP4 may also be causing this to occur [38]. Therefore, with CKAP4 possibly affecting numerous aspects in AKI and CKD pathology, this SASP protein may be of high interest for future investigation.

Cells that undergo senescence secrete a variety of inflammatory and stromal regulators collectively known as senescence-associated secretory phenotypes (SASPs) [39,40,41]. SASPs are suggested to help immune clearance and elimination of senescent cells [42]. However, SASPs can also adversely impact neighboring cells, extracellular matrices, and other structural components, resulting in chronic inflammation and leading to the initiation of senescence in healthy cells and vulnerable tissue [43,44,45,46,47]. The mechanisms involved in kidney disease are still largely unknown; however, there is growing evidence that the reduced regenerative capacity of the kidneys is associated with cellular senescence [48]. Pro-inflammatory and pro-fibrotic senescence-associated secretory phenotypes (SASPs), including TNFα, IL-6, IL-1B, and MCP-1 have been shown to be measurable in mice 24 h post-AKI [40,49]. Abnormal kidney repair, a maladaptive response following AKI, can lead to tubular epithelial cells assuming a senescence-like phenotype, and with the downregulation of Klotho expression, increased expression of cyclin kinase inhibitors and telomere shortening can occur [50]. This results in sustained secretion of profibrotic cytokines that can lead to fibrosis post-AKI and drive the kidney toward CKD [51].

PTX3, a member of the pentraxins superfamily, is shown to play a role in innate immunity, inflammation, and tissue remodeling, being mediated by toll-like receptors (TLR) [52,53,54,55]. High levels of PTX3 have been shown to be associated with worse outcomes in other organs including the brain following a stroke [56], and with low levels have reported to increase tumor development risk [57]. PTX3, a well-established senescence protein, is a multifunctional protein that plays complex regulatory roles in extracellular organisation and has been shown to increase dramatically in inflammatory conditions [58]. In cell models, PTX3 has been shown to stabilise the mitochondrial membrane potential and to suppress apoptosis, with levels shown to increase following ischemic reperfusion injury (IRI) and to be positively correlated with injury severity [59,60].

Mice deficient of PTX3 were shown to have worse recovery post-IRI compared to wild-type controls, and when mice were injected with PTX3 post-IRI, they had improved recovery compared to mice who were not, suggesting that PTX3 increases recovery in renal tubular cells post-injury [61,62]. Clinical studies have shown PTX3 levels to be significantly higher in AKI patients post-AKI than in non-AKI patients and higher in CKD patients compared to controls [63,64,65]. This may suggest that the high PTX3 levels seen in AKI and CKD patients are a protective mechanism aimed at protecting the kidneys post-injury. Furthermore, other clinical studies have shown PTX3 levels to be upregulated in CVD and diabetic patients [66,67]. In our study, however, where the control cohort included CVD and diabetic patients, PTX3 levels remained significantly upregulated in AKI and CKD when compared to controls. This highlights the extent to which PTX3 is upregulated in AKI and CKD and possibly highlights it as a biomarker of interest for further investigation for disease-risk stratification.

If promptly treated, AKI can resolve and the kidneys can recover to baseline function; however, the risk of progression to CKD is greatly increased following AKI [68,69]. The progression to CKD, following AKI, regardless of underlying cause, involves multiple mechanisms including immune cells, proximal tubular cells, and fibroblasts, where inflammation, hypoxia, and nephron loss occur [4]. AKI survivors are said to have progressed to CKD when their kidney function has remained permanently below their baseline function for 90 days [70]. A large meta-analysis reported 26% of patients who experienced AKI went on to develop CKD and 9% progressed to end-stage renal disease where renal replacement therapy was required [6]. A longitudinal study involving ICU patients who were followed up after 90 days reported that 25% of AKI patients progressed to CKD, with another reporting that 43.5% of hospitalised AKI patients progressed to CKD at 36-month follow-up [71]. Therefore, in addition to its ability to potentially aid in the diagnosis of AKI and CKD, CKAP4 and PTX3 may be able to help stratify AKI patients at higher risk of progression to CKD.

## 5. Limitations

Although this is the first type of study to use UHCs as controls for CKD and AKI, our approach may have pitfalls. For example, most diseases exist as comorbidities. There is a clear link between diabetes and CVD and kidney diseases. Our criteria may still include CVD and diabetes patients who may have underlying kidney dysfunction. An external study with a higher number of patients is needed to validate our results. Furthermore, this study is restricted to only CKD and AKI investigations, but the role of senescence biomarkers in different AKI/CKD aetiologies, for example, in glomerular diseases, needs to be investigated in further studies.

## 6. Conclusions

Senescence proteins CKAP4 and PTX3 were shown to be capable of distinguishing AKI and CKD patients from a comorbid control cohort including patients with diabetes, CVD, and RA. This suggests that senescence plays a role in both AKI and CKD pathology and that these senescence proteins may be useful for disease diagnosis. Furthermore, CKAP4 and PTX3 may be useful for the stratification of AKI/CKD patient progression risk.

## Figures and Tables

**Table 1:** Demographics for AKI patients, CKD patients and UHC.

Label	Levels	AKI	CKD	UHC	*p*-Value
Gender	Female	23	58	53	
	(%)	(53.5)	(37.4)	(53.0)	0.024
	Male	20	97	47	
	(%)	(46.5)	(62.6)	(47.0)	
BMI	Mean	30.1	29.3	30.7	
	(SD)	(10.1)	(5.5)	(7.7)	0.306
Age	Mean	60.5	58.7	59.5	
	(SD)	(14.8)	(17.3)	(12.9)	0.788

This table shows the demographics for AKI patients (*n* = 43), CKD patients (*n* = 155), and UHCs (*n* = 100). All AKI and CKD patients were age, sex, and BMI matched to UHC. Levels of significance are represented with *p*-values. No urea or CRP data were available for UHCs so no comparison could be completed.

## Data Availability

The raw data supporting the conclusions of this article will be made available by the authors on request.

## Supplementary Materials

The following supporting information can be downloaded at: https://www.mdpi.com/article/10.3390/cells13191613/s1, Supplementary Table S1, Comorbidities of AKI/CKD Patients.
