# Supplementary material for: Senescence Biomarkers CKAP4 and PTX3 Stratify Severe Kidney Disease Patients
Source: Cells. 2024 Sep 26;13(19):1613. doi: 10.3390/cells13191613 (PMC11475272; doi:10.3390/cells13191613)
Supplement: Supplementary file 1 [file cells-13-01613-s001.zip › cells-3157932-supplementary.pdf]

Supplementary Table 1

Co-Morbidities	Status	AKI	CKD	p-value
Diabetes	No	37 (86.0)	149 (96.1)	0.037
	Yes	6 (14.0)	6 (3.9)	
Hypercholesterolemia	No	29 (67.4)	99 (63.9)	0.800
	Yes	14 (32.6)	56 (36.1)	
Myocardial Infarction	No	36 (83.7)	144 (92.9)	0.120
	Yes	7 (16.3)	11 (7.1)	
Stroke	No	40 (93.0)	143 (92.3)	1.000
	Yes	3 (7.0)	12 (7.7)	
CVD	No	30 (69.8)	124 (80.0)	0.222
	Yes	13 (30.2)	31 (20.0)	
Liver Disease	No	37 (86.0)	149 (96.1)	0.037
	Yes	6 (14.0)	6 (3.9)	
